# Associated factors of cardiac valve calcification and its prognostic effects among patients with chronic kidney disease: a systematic review and meta-analysis

**DOI:** 10.3389/fcvm.2023.1120634

**Published:** 2023-04-26

**Authors:** Jialing Zhang, Qi Pang, Shiyuan Wang, Leiyun Wu, Aihua Zhang

**Affiliations:** ^1^ Department of Nephrology, Xuanwu Hospital, Capital Medical University, Beijing, China; ^2^ National Clinical Research Center for Geriatric Disorders, Xuanwu Hospital, Capital Medical University, Beijing, China

**Keywords:** cardiac valve calcification, cardiovascular mortality, chronic kidney disease, mortality, risk factor

## Abstract

**Background:**

Cardiac valve calcification (CVC) is highly prevalent and a risk factor for adverse outcomes in patients with chronic kidney disease (CKD). This meta-analysis aimed to investigate the risk factors for CVC and association between CVC and mortality in CKD patients.

**Method:**

Three electronic databases including PubMed, Embase, and Web of Science were searched for relevant studies up to November 2022. Hazard ratios (HR), odds ratios (OR), and 95% confidence intervals (CI) were pooled using random-effect meta-analyses.

**Results:**

22 studies were included in the meta-analysis. Pooled analyses showed that CKD patients with CVC were relatively older, had a higher body mass index, left atrial dimension, C-reaction protein level, and a declined ejection fraction. Calcium and phosphate metabolism dysfunction, diabetes, coronary heart disease, and duration of dialysis were all predictors for CVC in CKD patients. The presence of CVC (both aortic valve and mitral valve) increased the risk of all-cause and cardiovascular mortality in CKD patients. However, the prognostic value of CVC for mortality was not significant anymore in patients with peritoneal dialysis.

**Conclusion:**

CKD patients with CVC had a greater risk of all-cause and cardiovascular mortality. Multiple associated factors for development of CVC in CKD patients should be taken into consideration by healthcare professionals to improve prognosis.

**Systematic Review Registration:**

https://www.crd.york.ac.uk/PROSPERO/, identifier [CRD42022364970].

## 1. Introduction

Chronic kidney disease (CKD), defined as the occurrence of renal structural alteration and dysfunction more than three months, affects approximately 15% of the adult population worldwide, which puts a huge burden on public health (1). Cardiovascular diseases (CVD) remain a most serious complication to CKD, causing an increasing morbidity and mortality for patients with CKD (2–4). Accumulating evidence suggested that vascular calcification is one of the major causes of CVD in CKD patients (5), and represents a strong predictor for mortality (6–8).

Patients with CKD exhibit a higher cardiovascular risk compared to non-CKD cohort (9). Non-traditional risk factors should be emphasized to identify the mechanism of accelerated atherosclerosis, apart from traditional risk factors, such as age, male, hypertension, hypercholesterolemia and obesity. The Kidney Disease Improving Global Outcome (KDIGO) Guideline suggested that cardiac valve calcification (CVC) should be emphasized in the risk stratification of CVD in CKD patients (10).

Uremic toxins might be a key regulator of calcification for end-stage renal disease patients (11). Numerous studies reported a highly prevalence of CVC and stenosis development in patients undergoing renal replace therapy (12). Aortic valve calcification and aortic stenosis may occur in the early stages of kidney failure in a GFR-dependent manner (13, 14). CVC was recognized as a contributor to all-cause and cardiovascular mortality in hemodialysis patients (15, 16), however, the prognostic value was not reliable (17, 18). Multiple risk factors for CVC have been explored, such as traditional factors (age, hypertension, and diabetes) and non-traditional factors (hyperphosphatemia, calcium phosphate product, fibroblast growth factor 23, inflammation and malnutrition) (19–21). It is crucial to identify independent risk factors for CVC in CKD patients. However, the pathophysiology of CVC is not completely understood.

To our knowledge, analysis of the impact of CVC on mortality of CKD patients has not yet been performed. The aim of the meta-analysis was to investigate the clinical prognosis of CVC, in addition to the associated factors, in patients with CKD.

## 2. Method

### 2.1. Search strategies

We conducted a systematic review, prospectively registered on PROSPERO (ID: CRD42022364970). This systematic review was performed based on the Preferred Reporting Items for Systematic Reviews and Meta-Analysis (PRISMA) guidelines (22). We searched the PubMed, Embase, and Web of Science database up to November 2022. The search strategies were shown in Supplementary Material. The references of selected articles were searched manually for additional eligible studies.

### 2.2. Study selection

Two researchers screened all abstracts to verify potentially relevant articles for this review. Any disagreement was settled by another independent reviewer. Studies met the following criteria were eligible: (1) involving participants with CKD regardless of dialysis; reporting the association of CVC and mortality, including associated risk factors of CVC; (2) reporting of the hazard ratio (HR) or odds ratio (OR) with 95% confidence interval (CI); (3) cohort studies (prospective or retrospective). CVC was defined as bright echoes on one or more cusps of more than 1 mm in either mitral or aortic valves or both by echocardiographs. All echocardiographic data were acquired according to the guidelines of the American Society of Echocardiography (23). Studies without adjustment for specific potential confounders and non-English studies were excluded.

### 2.3. Data extraction and statistical analyses

Data were collected by two authors from the eligible studies, including first author's last name, publication year, country, study design, demographic characteristics of patients and outcomes. All statistical analyses were conducted in Review Manager 5.3 software. Binomial factors are presented as OR with 95% CI, while continuous variables are presented as standardized mean difference (SMD) with 95% CI. HR adjusted for confounding variables and 95% CI were extracted from included studies in terms of all-cause mortality, CV mortality and CV events. Statistical heterogeneity among studies was evaluated using the *I*^2^ index (24). A random-effect model was applied to due to a significant heterogeneity, otherwise a fixed-effect model was applied. The Newcastle–Ottawa Scale (NOS) for cohort studies was applied to assess the quality of cohort study (25). Subgroup analysis was performed according to the type of cardiac valve and dialysis modality. Publication bias was explored by Egger test. *P* < 0.05 was considered to be statistically significant.

## 3. Result

### 3.1. Baseline characteristics of the included studies

**Figure 1:**
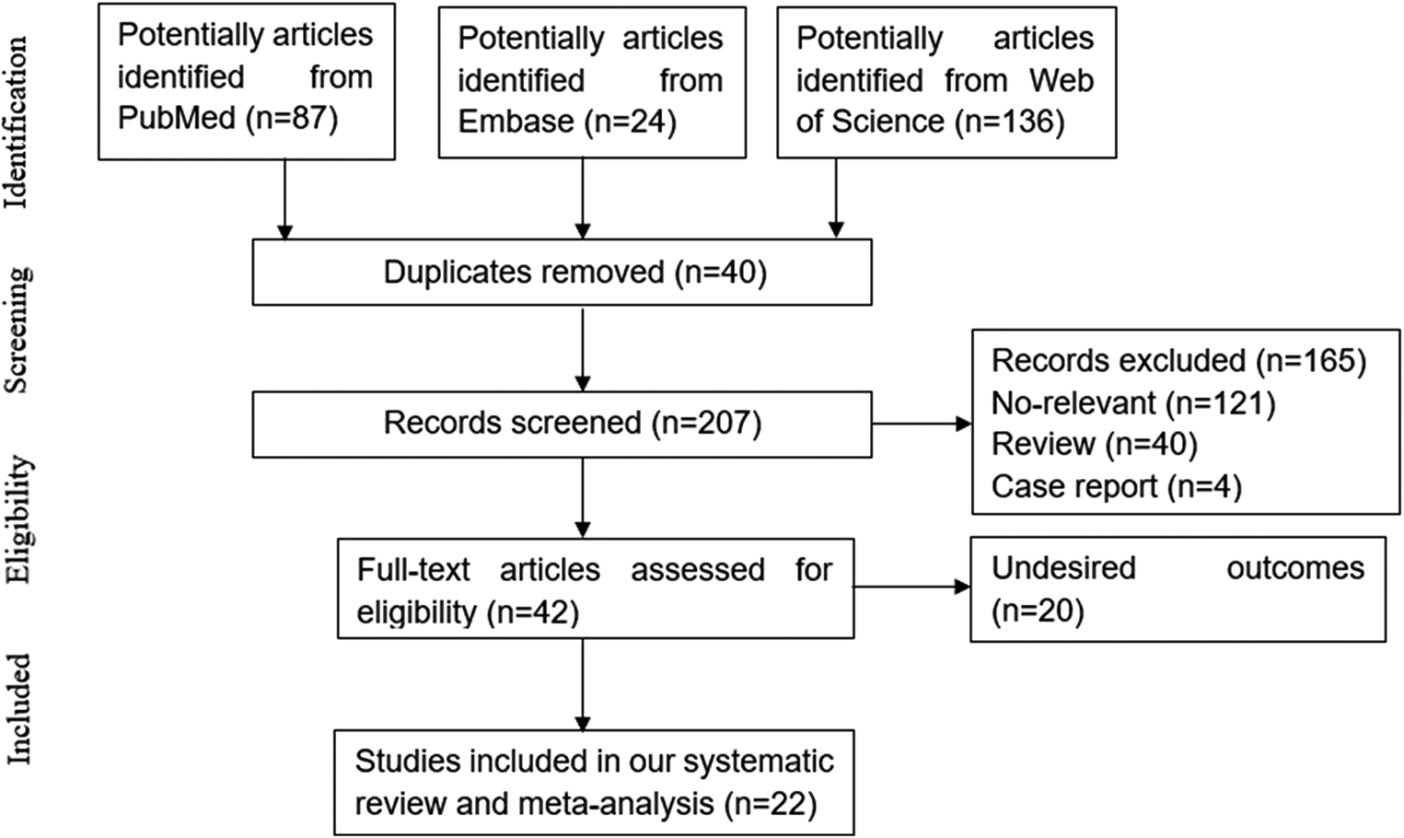
Flow chart of article selection.

Following the inclusion criteria, 22 (12, 15–18, 26–42) articles were eventually included in our study. The study selection process is depicted in Figure 1. Among those including articles, 13 studies only reported associated factors for development of CVC, while the other 9 (15–18, 38–42) studies also explored the association of CVC and mortality meanwhile. Patients treated with hemodialysis were enrolled in 9 studies, while patients treated with peritoneal dialysis were enrolled in 6 studies. 3 studies only included patients with non-dialysis CKD, while 2 involved both hemodialysis and peritoneal dialysis patients. The basic characteristics of the included studies at baseline are listed in Table 1.

**Table 1:** Characteristics of studies included.

Study	Country	Study design	Cohort	Sample size	Definition of valve	Type of cardiac valve	Age (year)	Male (%)	Duration of dialysis	Diabetes (%)	Coronary heart disease (%)	Left ventricular ejection fraction (%)	Outcome	NOS
Kim, I. Y (2022)	Korea	Retrospective	CKD without dialysis	513	Two-dimensional echocardiography	Aortic valve calcification and mitral valve calcification		263		264 (51.5)	111 (21.6)		Presence of valve calcification	6
Usuku, H (2019)	Japan	Retrospective	HD	95	Transthoracic echocardiography	Mitral valve calcification	Presence: 65.0 ± 10.7; Absence: 62.6 ± 13.2	51	Presence: 13.4 ± 8.6 years; Absence: 7.7 ± 8.4 years	23 (24.2)	9 (9.5)	Presence: 60.1 ± 11.5; Absence: 62.3 ± 8.3	Presence of valve calcification	6
Torun, D (2005)	Turkey		HD	79	Two-dimensional echocardiography	Aortic valve calcification and mitral valve calcification	52.2 ± 13.6	42	Presence: 49 (27–99) months; Absence: 26 (17–52) months	28 (35.4)			Presence of valve calcification	6
Wang, A. Y. M (2001)	China	Retrospective	CAPD	137	Echocardiography	Cardiac valve calcification	56 (22–77)	65	45 (5–151) months	40 (29.2)			Presence of valve calcification	6
Li, B (2022)	China	Retrospective	non-dialysis CKD stage 3–5	483	Echocardiography	Cardiac valve calcification	Presence: 71.65 ± 10.50; Absence: 56.08 ± 14.97	267		148 (30.6)			Presence of valve calcification	6
Unal, H. U (2014)	Turkey	Retrospective	CKD	60	Transthoracic echocardiography	Mitral valve calcification	Presence: 41.93 ± 15.83; Absence: 42.87 ± 16.41	41 (68.33)		13 (21.7)	4 (6.7)	Presence: 64.86 ± 5.49; Absence: 65.29 ± 4.56	Presence of valve calcification	6
Sharma, R (2007)	UK	Prospective	ESRD (Predialysis 61 (44%); HD 50 (36%); CAPD 29 (20%))	140	Transthoracic echocardiography	Mitral valve calcification	52 ± 13	90	2.61 ± 1.48 months	54 (38)	40 (29)	Presence: 62 ± 15; Absence: 72 ± 12	Presence of valve calcification	6
Wang, L (2021)	China	Prospective	CKD1–4	2,756	Echocardiography	Aortic valve calcification and mitral valve calcification	48.73 ± 13.73	1,613 (58.53)		470 (21.03)	243 (10.91)		Presence of valve calcification; Mortality and CV events	7
Abd Alamir, M (2015)	USA	Retrospective	CKD	2,070	Computed tomography scan	Mitral valve calcification	Presence: 64.7 ± 7.7; Absence: 56.7 ± 11.6	1,112 (53.7)		972 (47)	305 (14.7)		Presence of valve calcification	6
Xiong, J. Q (2022)	China	Cross-sectional	HD and hemodiafiltration	293	Echocardiography	Aortic valve calcification and mitral valve calcification	64 ± 7.2	160	Presence: 32 (8- 72) months; Absence: 19 (7- 43) months	142 (48.5)			Presence of valve calcification	6
Rebić, D (2015)	Bosnia and Herzegovina	Prospective	PD	50	Two-dimensional echocardiography	Cardiac valve calcification	60.5 (19–76)	25 (50)		26 (52)		Presence: 48.38 ± 6.9; Absence: 62.37 ± 8.2	Presence of valve calcification	6
Sánchez-Perales, C (2015)	Spain		Dialysis (83% HD, 17% PD)	256	Doppler-echocardiography	Cardiac valve calcification	65.1 ± 15.9	146		73 (28.5)		Presence: 64.6 ± 6.9; Absence: 65.5 ± 8.8	Presence of valve calcification	6
Wang, C (2013)	China	Observational	PD	117	Echocardiography	Aortic valve calcification and mitral valve calcification	56.9 ± 15	71	31 (24–40) months	32 (27.4)	11 (9.4)		Presence of valve calcification	6
Tian, Y (2016)	China	Observational	PD	194	Echocardiography	Cardiac valve calcification	60.5 ± 13	105 (54.1)	Presence: 24.3 ± 10.5 months; Absence: 19.5 ± 10.0 months				New-onset valve calcification	6
Raggi, P (2011)	Atlanta	Prospective	HD	144	Two-dimensional echocardiograph	Aortic valve calcification and mitral valve calcification	55.4 ± 14.6	(49.3)	20.9 ± 10.4 months	52 (36.1)			Mortality	6
Chen, X. N (2015)	China		HD	110	Two-dimensional echocardiography	Cardiac valve calcification	55.2 ± 1.4	64 (58.2)	29.85 (3.00–225.5) months	15 (13.6)			Mortality and CV mortality	7
Yang, X (2018)	China	Prospective	PD	324	Two-dimensional echocardiography	Cardiac valve calcification	57.4 ± 14.1	166 (51)	31.5 (12.4–57.1) months	84 (25.9)	99 (30.6)		Mortality and CV events	7
Bai, J (2022)	China	Retrospective	HD	434	Doppler-echocardiography	Aortic valve calcification and mitral valve calcification	Presence: 56.81 ± 11.78; Absence: 49.74 ± 13.87	270 (62.2)	Presence: 3.58 ± 3.19 years; Absence: 3.29 ± 3.33 years	102 (23.5)	240 (55.3)	Presence: 61 ± 11; Absence: 62 ± 8	Mortality and CV mortality	7
Lin, F. J (2019)	China	Prospective	HD	174	Comprehensive echocardiography	Aortic valve calcification and mitral valve calcification	Presence: 60.64 ± 12.13; Absence: 51.70 ± 12.4	109		28 (16.1)	14 (8)	AVC Presence: 66.12 ± 5.97; Absence: 66.01 ± 5.17; MVC Presence: 65.78 ± 5.29; Absence: 66.13 ± 5.50	CV events	6
Panuccio, V (2004)	Italy		HD	202	Doppler-echocardiography	Cardiac valve calcification	59 ± 15	113 (55.9)	Presence: 61 (30–120) months; Absence: 38 (18–96) months	27 (13.4)			Mortality and CV mortality	7
Li, M (2020)	China	Retrospective	HD	183	Doppler-echocardiography	Aortic valve calcification and mitral valve calcification	56.1 ± 17.0	104 (56.8)	Presence: 22.8 (10.2, 59.4) months; Absence: 11.0 (6.2, 35.6) months	59 (32.2)	43 (23.5)	AVC Presence: 61.6 ± 10.0; Absence: 64.8 ± 7.8; MVC Presence: 64.6 ± 6.6; Absence: 63.6 ± 9.0	Mortality and CV mortality	7
Shen, A (2021)	China	Retrospective	PD	310	Echocardiography	Cardiac valve calcification	57 ± 15.9	179	Presence: 31.2 ± 26.9 months; Absence: 37.6 ± 29.0 months	45 (14.5)		Presence: 61.85 ± 9.11; Absence: 60.77 ± 7.97	Mortality	6

CKD, chronic kidney disease; HD, hemodialysis; PD, peritoneal dialysis; ESRD, end-stage renal disease; AVC, aortic valve calcification; MVC, mitral valve calcification.

**Table 2:** Risk factors for CVC in patients with CKD.

Factor	Odds ratio	95% confidence interval	*p*	*I*^2^(%)
Age	1.09	1.06, 1.12	<0.00001	74
Male	0.97	0.71, 1.32	0.83	0
Albumin	0.91	0.83, 1.01	0.08	69
Hemoglobin	0.93	0.8, 1.08	0.37	77
Ca*P product	1.2	1.08, 1.34	0.001	87
CRP	1.07	1.01, 1.13	0.02	56
PTH	1.05	0.97, 1.14	0.25	78
Diabetes	1.7	1.35, 2.14	<0.0001	45
Hypertension	1.17	0.55, 2.47	0.68	69
Coronary heart disease	2.96	1.27, 6.92	0.01	68
Duration of dialysis	1.08	1.01, 1.16	0.03	68

Ca, calcium; P, phosphate; CRP, C-reaction protein; PTH, parathyroid hormone; CVC, cardiac valve calcification.

### 3.2. Comparing basic demographic and echocardiography characteristic between CKD patients with and without CVC

Advanced age (SMD: 9.35, 95% CI: 6.45, 12.25), higher BMI (SMD: 0.85, 95% CI: 0.17, 1.53), and higher systolic blood pressure (SMD: 9.31, 95% CI: 4.58, 14.05) were significantly associated with a development of CVC in CKD patients. Studies also evaluated several echocardiographic markers, including left atrial dimension, left ventricular ejection fraction (LVEF), early diastolic transmitral flow velocity to atrial contraction transmitral flow velocity ratio (*E*/*a* ratio), and early diastolic transmitral flow velocity to diastolic early mitral annular velocity ratio (*E*/*e*′ ratio). CKD cohorts with CVC were more likely to be accompanied with a higher *E*/*e*′ ratio (SMD: 2.97, 95% CI: 0.31, 5.62) by meta-analysis of three studies, and higher left atrial dimension (SMD: 2.15, 95% CI: 1.01, 3.29) by meta-analysis of five studies. Four studies reported a lower E/a ratio (SMD: −0.17, 95% CI: −0.25, −0.09) while nine studies reported a lower LVEF (SMD: −2.19, 95% CI: −4.06, −0.32) in patients with CVC (shown in Supplementary Figure S1).

### 3.3. Basic characteristics and comorbidities predictors for CVC in patients with CKD

**Figure 2:**
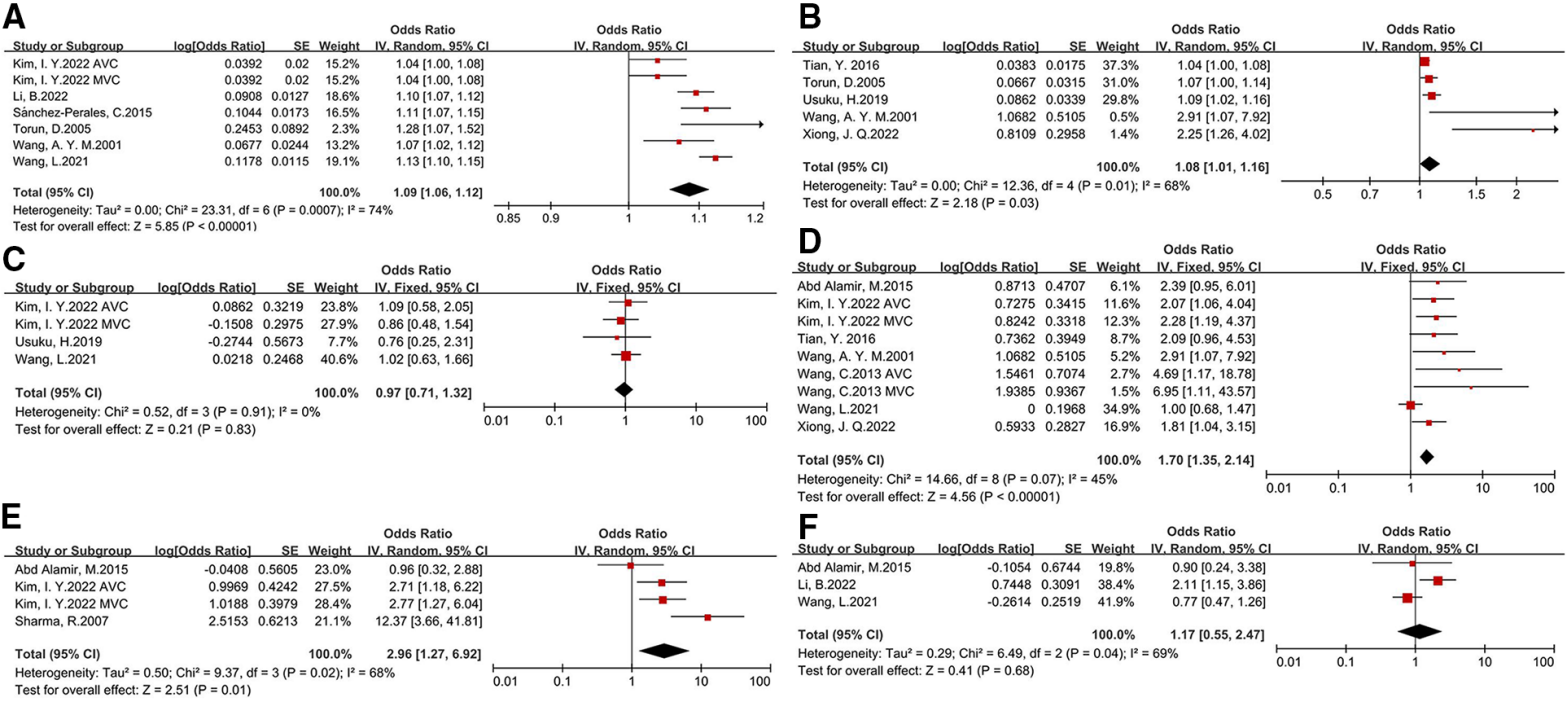
Basic characteristics and comorbidity risk factors for development of CVC in CKD patients [(**A**) age, (**B)** duration of dialysis, (**C**) male sex, (**D**) diabetes, (**E**)coronary heart disease, (**F**) hypertension].

We compared demographic characteristics between CKD patients with and without CVC. Older age (OR: 1.09, 95% CI: 1.06–1.12), and longer duration of dialysis (OR: 1.08, 95% CI: 1.01–1.16) were significantly associated with the presence of CVC. CKD patients with diabetes as shown in Figure 2, coronary heart disease (OR:2.96, 95% CI: 1.27-6.92) (OR: 1.7, 95% CI: 1.35–2.14), coronary heart disease (OR: 1.09, 95% CI: 1.06–1.12) had a higher risk for development of CVC. The effect of male sex and hypertension on CVC in CKD patients was not significant (asshown in Figure 2 and Table 2).

### 3.4. Laboratory tests predictors for CVC in patients with CKD

**Figure 3:**
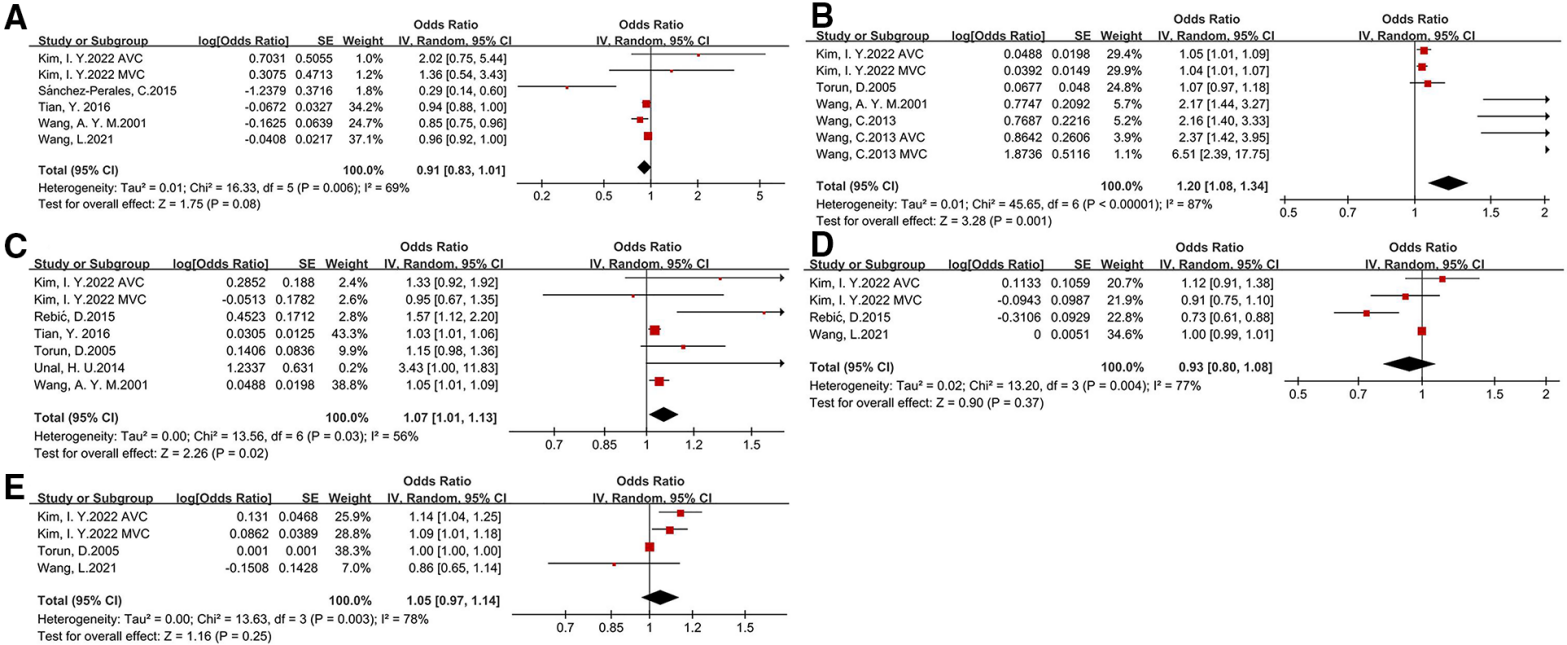
Laboratory risk factors for development of CVC in CKD patients [(**A**) albumin, (**B**) calcium*phosphate, (**C**) C-reaction protein, (**D**) hemoglobin, (**E**)parathyroid hormone].

We investigated several laboratory examinations of CKD patients in this meta-analysis (as shown in Figure 3 and Table 2). The results suggested that an increased calcium*phosphate product (OR: 1.2, 95% CI: 1.08–1.34) is associated with a higher risk of CVC. The association of parathyroid hormone and CVC was slight. There is a tight link among malnutrition, inflammation and atherosclerosis (MIA) in CKD patients. We only found C-reaction protein (CRP) (OR: 1.07, 95% CI: 1.01–1.13), but not albumin and hemoglobin, is associated with a higher risk of CVC. Besides, the level of total cholesterol, low-density lipoprotein cholesterol, high-density lipoprotein cholesterol, and triglyceride between patients with CVC and without CVC was relatively comparable (shown in Supplementary Figure S2).

### 3.5. Association between CVC and all-cause mortality and cardiovascular mortality

**Figure 4:**
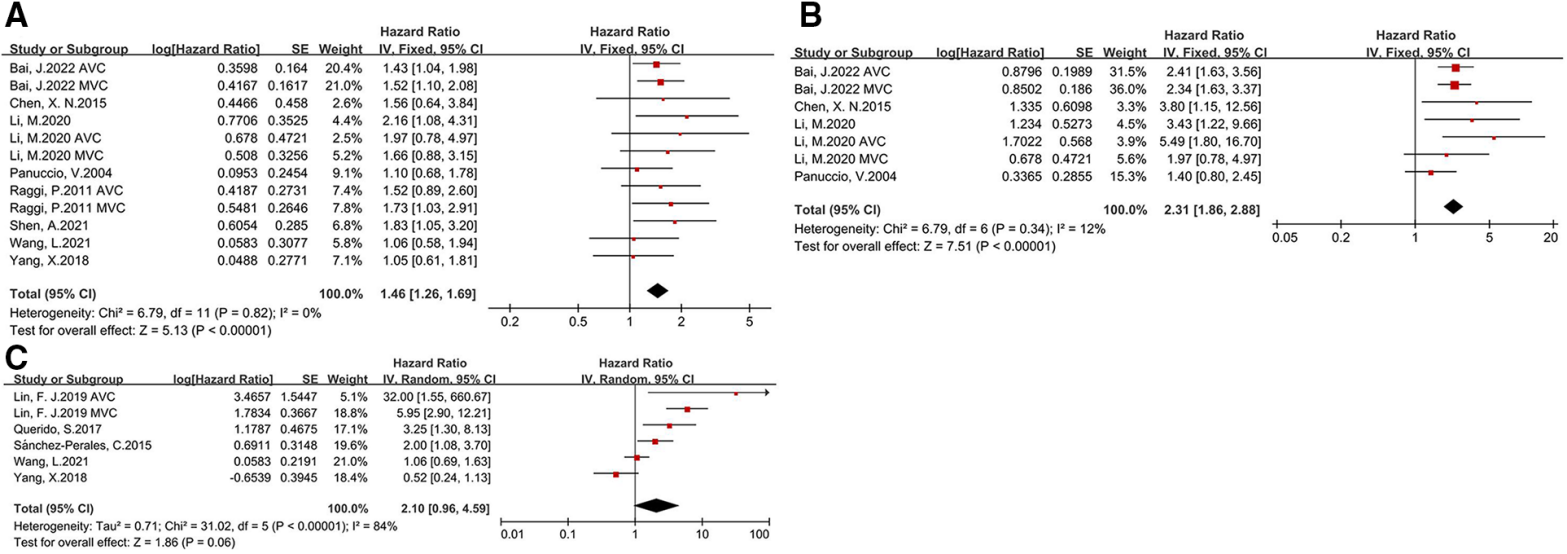
Forest plot of clinical outcomes for CKD patients with CVC [(**A**) all-cause mortality, (**B**) cardiovascular mortality, (**C**) cardiovascular events].

The relationship between CVC and prognosis in CKD patients were assessed covering all-cause deaths (8 studies), cardiovascular deaths (4 studies), and cardiovascular events (5 studies). Upon meta-analysis of including studies, we found that the presence of CVC was associated with greater risk of all-cause mortality (HR: 1.46, 95% CI: 1.26–1.69) and cardiovascular mortality (HR: 2.31, 95% CI: 1.86–2.88) (as shown in Figure 4). All studies included in the analysis utilized a fixed effects model because of low heterogeneity (*I*^2^ = 0%, *I*^2 ^= 12%, respectively). Nonsignificant publication bias was examined with Egger test (*p* = 0.535). However, the effect of CVC and cardiovascular events was slight in CKD patients (HR: 2.1, 95% CI: 0.96–4.59). Due to the small number of studies addressing other clinical outcomes, subgroup analysis was only performed for the association of CVC and all-cause mortality. By subgroup analysis according to the different type of valve calcification, we found significant association between valve calcification and all-cause mortality in both aortic valve (HR: 1.49, 95% CI: 1.15–1.94) and mitral valve (HR: 1.59, 95% CI: 1.24–2.03). However, when stratified by the patients type, the predictive value of CVC for mortality was significant only in hemodialysis patients (HR: 1.51, 95% CI: 1.29–1.78), but not peritoneal dialysis patients (HR: 1.38, 95% CI: 0.93–2.03) (as shown in Supplementary Material).

## 4. Discussion

Our findings demonstrated that CVC increased the risk of all-cause mortality and cardiovascular mortality in patients with CKD, regardless of aortic valve or mitral valve. Various factors were firstly reported to be probably related to the development of CVC, including age, comorbidities (coronary heart disease and diabetes), CRP and calcium-phosphate metabolism.

Vascular calcification is defined as a form of calcium-phosphate complexes deposition in the vasculature, mainly including intimal calcification, Mönckeberg medial arterial calcification, and valvular calcification (43). The echocardiographic evaluation of CVC is recommended as a routine examination by the KDIGO guidelines (44). CVC is increasingly common in dialysis patients (45). However, the predictive value of calcified valves for mortality remains controversial (46).

By meta-analysis, we found CKD patients with CVC were at a higher hazard for all-cause mortality and cardiovascular mortality, which is similar to another meta-analysis (47). However, in our study, we included more updated articles and found the effect of CVC on cardiovascular events was limited, probably due to a different definition of cardiovascular events in individual study. CVC is more frequently observed as the decline of residual renal function, including both aortic valve calcification and mitral valve calcification (48). In a multicenter study of stage 5 CKD patients, mitral valve calcification remained associated with all-cause mortality after adjusting for confounding, while aortic valve calcification was not (15). In our subgroup analysis, we found both aortic valve and mitral valve calcification could predict all-cause mortality in CKD patients, effectively. The mitral valve calcification may lead to mitral regurgitation and stenosis, whereas aortic valve calcification is more likely to cause aortic stenosis. Nevertheless, the predictive value of CVC was obvious only in hemodialysis patients, but not peritoneal dialysis patients, which is not consistent with the results of Wang, Z (47). In another publication involving peritoneal dialysis patients, the significance of CVC associated with all-cause mortality was lost when adjusting for covariates (39). Valve calcification was more frequently found in hemodialysis compared to peritoneal dialysis patients (49). The residual renal function and the electrolyte imbalance in peritoneal dialysis patients might be different from that in hemodialysis patients. Peritoneal dialysis patients may have a greater time-averaged exposure to phosphate than hemodialysis (50). The calcium balance between hemodialysis and peritoneal dialysis needs to be further explored. On echocardiography, CVC patients had a higher left ventricle mass index, pulmonary artery pressure, and a lower ejection fraction (51). CVC has also been associated with markers of atherosclerosis and severity of coronary artery disease (52), including carotid intima-media thickness, and plaque in the carotid arteries (53, 54). CVC on routine echocardiography can be used for risk assessment in CKD patients to reduce cardiovascular events. Verifying the risk factors for CVC is important to improve prognosis.

The pathogenetic features including mechanical stress, metabolic disorder and inflammatory disturbances were described. However, the pathophysiology and clinical impact of CVC was still unknown. In this meta-analysis, we found CKD patients with CVC were older, with a lower ejection fraction and relatively worse diastolic function. Several diseases could lead to a progressive left atrial enlargement, involving heart failure and hypertension. CVC might alter volume overload to induce left atrial dysfunction. A large left atrial diameter was an independent risk factor for mortality in hemodialysis patients or renal allograft recipients (55, 56). Mitral valve replacement in patients with mitral valve stenosis could improve left atrial contractile function by inhibiting apoptotic process (57). *E*/*e* ratio was associated with mortality and cardiovascular hospitalization in patients with heart failure with preserved ejection fraction (58). A review also confirmed that an increased *E*/*e* ratio was associated with an increased risk for chronic kidney disease progression (59). In patients with aortic valve stenosis, LVEF were significantly lower than that of patients with normal aortic valve (60). Left ventricular diastolic dysfunction was another predictor for all-cause mortality and cardiovascular outcomes in pre-dialysis CKD patients, while a stringent linear correlation was also clarified between *E*/*e* ratio and mortality (61). Our study corroborates the notion that several echocardiography parameters could be a marker of CVC in CKD patients.

Diabetes and hypertension are common causes of CKD. Diabetic nephropathy probably had an independent effect on *E*/*e* ratio, left ventricular diastolic dysfunction and cardiac valve regurgitation (62). In addition, diabetes patients are with a higher coronary artery calcification (CAC) score, and incidence rate of major adverse cardiovascular events (63). A higher CAC score was significantly associated with fibroblast growth factor 23, parathyroid hormone, and inflammation index (64). High glucose conditions can exacerbate oxidative stress and calcification through the induction of CD36 scavenger receptors (65) and endothelial dysfunction (66). The alteration involved several pathways activated by hyperglycemia advanced glycation-end products.

Chronic kidney disease-mineral and bone disease, a common complication of CKD, is shown as biochemical abnormalities of calcium, phosphate, vitamin D, parathyroid hormone (PTH), bone disorders, and calcification. In our study, we found higher level of Ca*P product was a significant risk factor for CVC in CKD patients, although the predictive value of PTH was weak. Aortic valve calcification might be mediated through a process of osteoblast-like differentiation (67). Evidence showed that hyperphosphatemia, hypercalcemia could promote osteogenic/chondrogenic differentiation, apoptosis of VSMC (6). An elevated Ca concentration probably stimulate VSMC mineralization by elevating Ca*P product (68). Considering the number of studies exploring the association between serum Ca, serum P and CVC was small, we could not perform a meta-analysis in this study. Meaningfully, we prompted that imbalanced Ca and P metabolism probably contributes to CVC.

CKD patients are under a chronic low-grade inflammatory and malnutrition state, resulting in a higher risk of morbidity and mortality (69). The term MIA syndrome has been proposed. In our study, CRP was a predictor for CVC in patients with CKD. Chronic inflammation with activation of CRP and proinflammatory cytokines is related to an aggravated oxidative stress and endothelial dysfunction. Various inflammatory biomarkers were significantly associated with arterial calcification in CKD patients (70), including β2-microglobulin, interleukin 2, interleukin 8 and interleukin 18. The increase in the concentration of CRP is proportional to the decrease of renal function (71) and progression of abdominal aortic calcification in hemodialysis patients (72). The activation of dendritic cell function by CRP might be involved during atherogenesis (73). However, the predictive value of albumin, hemoglobin, cholesterol, and triglyceride for CVC was poor in our meta-analysis. More index considering nutrition and inflammation should be addressed in the future study.

As far as we know, this is the first meta-analysis to explore risk factors for development of CVC in CKD patients. Our meta-analysis included relatively recent studies, and were larger and more extensive in number and risk factors. In addition, we confirmed that both aortic valve and mitral valve calcification could predict poor prognosis in CKD patients. Our findings suggested that a comprehensive evaluation of various risk factors and routine echocardiography should not be overlooked. Our study had some limitations. The adjust covariates were different in individual study, which might affect the stability of our study. Besides, the sample size of several including studies were relatively small, probably leading to a bias. Due to the limitation of the data from original articles, we could not explore the definite associated factors for calcified aortic valve and mitral valve, separately. Finally, the number of studies reporting the association of CVC and other outcomes was small, in particular coronary heart disease, stroke and cerebrovascular disease. More larger sample size clinical studies are preferred to further confirm the reliability of our results.

## 5. Conclusion

Our meta-analysis indicated that both aortic valve and mitral valve calcification could predict all-cause mortality and cardiovascular mortality in patients with CKD. Various risk factors should be considered to prevent development and progression of CVC, including advanced age, diabetes, coronary heart disease, CRP and calcium-phosphate metabolism disorder. Further research is required to determine the mechanisms for CVC increasing the risk of mortality in CKD patients through large multicenter studies.

## Data Availability

The original contributions presented in the study are included in the article/**Supplementary Material**, further inquiries can be directed to the corresponding author.
