# Supplementary material for: Associated factors of cardiac valve calcification and its prognostic effects among patients with chronic kidney disease: a systematic review and meta-analysis
Source: Front Cardiovasc Med. 2023 Apr 26;10:1120634. doi: 10.3389/fcvm.2023.1120634 (PMC10169583; doi:10.3389/fcvm.2023.1120634)
Supplement: Supplementary file 1 [file Datasheet1.pdf]

Supplementary Figure 1 Association of general and echocardiography characteristics between CKD patients with and without CVC (A. age, B. body mass index, C. systolic blood pressure, D. E/e ratio, E. E/A ratio, F. ejection fraction, G. left atrial dimension)

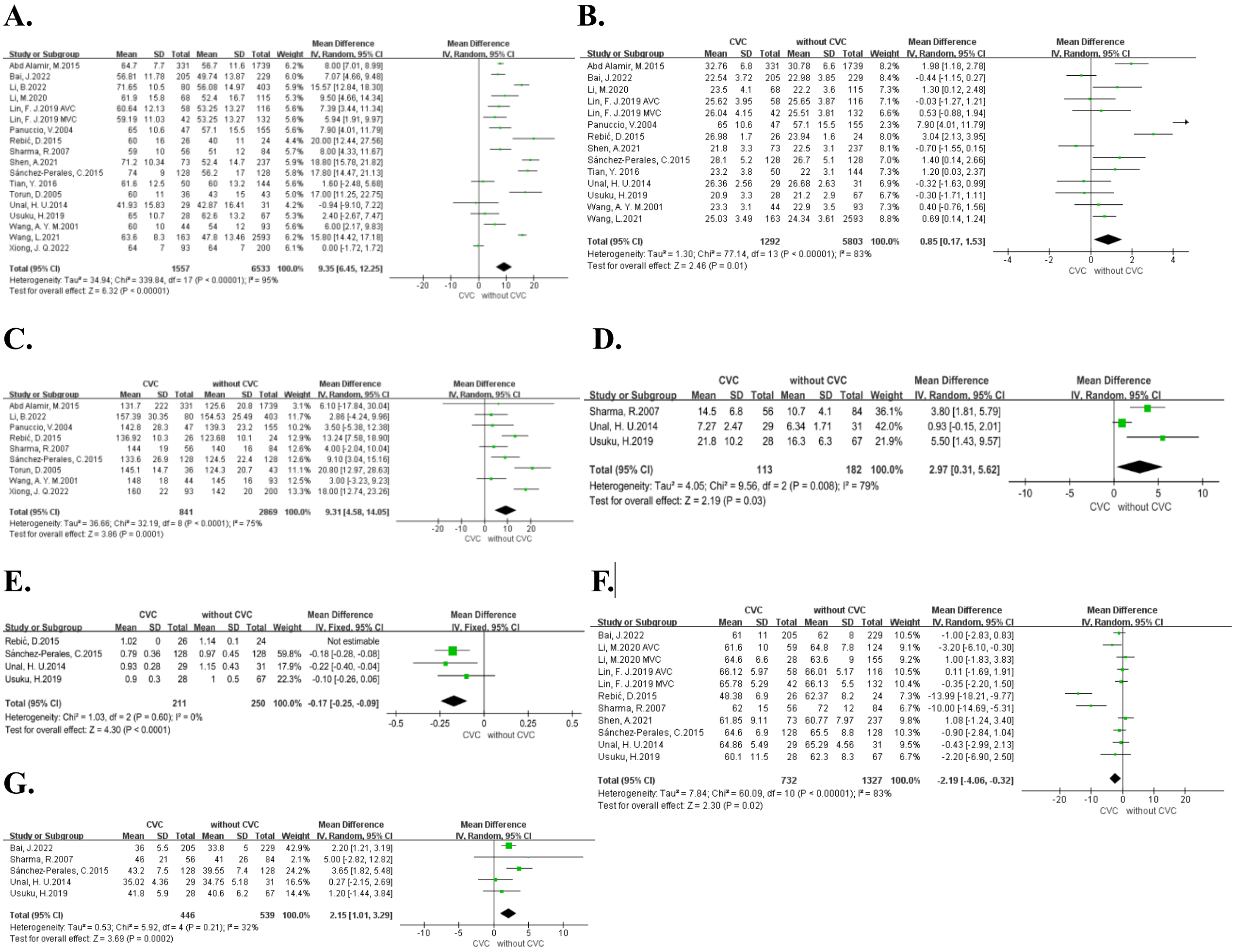

Supplementary Figure 2 Association of laboratory index between CKD patients with and without CVC (A. total cholesterol, B. low-density lipoprotein cholesterol, C. high-density lipoprotein cholesterol, D. triglyceride)

A.

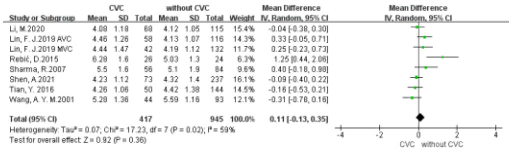

B.

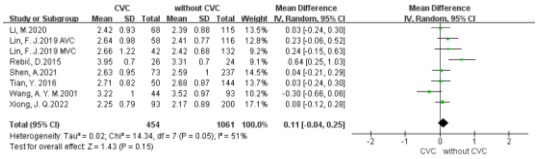

C.

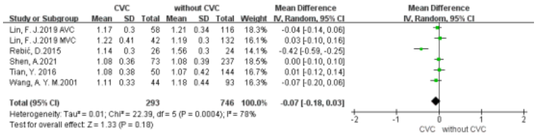

D.

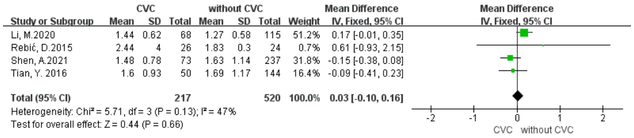

Supplementary Figure 3 Subgroup analysis for mortality according to the type of valve calcification

Study or Subgroup	log[Hazard Ratio]	SE	Weight	Hazard Ratio IV, Random, 95% CI
**26.2.1 aortic valve calcification**				
Bai, J.2022 AVC	0.3598	0.164	31.8%	1.43 [1.04, 1.98]
Li, M.2020 AVC	0.678	0.4721	3.8%	1.97 [0.78, 4.97]
Raggi, P.2011 AVC	0.4187	0.2731	11.5%	1.52 [0.89, 2.60]
**Subtotal (95% CI)**			**47.1%**	**1.49 [1.15, 1.94]**
Heterogeneity: Tau² = 0.00; Chi² = 0.41, df = 2 (P = 0.81); I² = 0%				
Test for overall effect: Z = 2.97 (P = 0.003)				
**26.2.2 mitral valve calcification**				
Bai, J.2022 MVC	0.4167	0.1617	32.7%	1.52 [1.10, 2.08]
Li, M.2020 MVC	0.508	0.3256	8.1%	1.66 [0.88, 3.15]
Raggi, P.2011 MVC	0.5481	0.2646	12.2%	1.73 [1.03, 2.91]
**Subtotal (95% CI)**			**52.9%**	**1.59 [1.24, 2.03]**
Heterogeneity: Tau² = 0.00; Chi² = 0.20, df = 2 (P = 0.90); I² = 0%				
Test for overall effect: Z = 3.63 (P = 0.0003)				
**Total (95% CI)**			**100.0%**	**1.54 [1.29, 1.85]**
Heterogeneity: Tau² = 0.00; Chi² = 0.72, df = 5 (P = 0.98); I² = 0%				
Test for overall effect: Z = 4.68 (P < 0.00001)				
Test for subgroup differences: Chi² = 0.11, df = 1 (P = 0.74), I² = 0%				

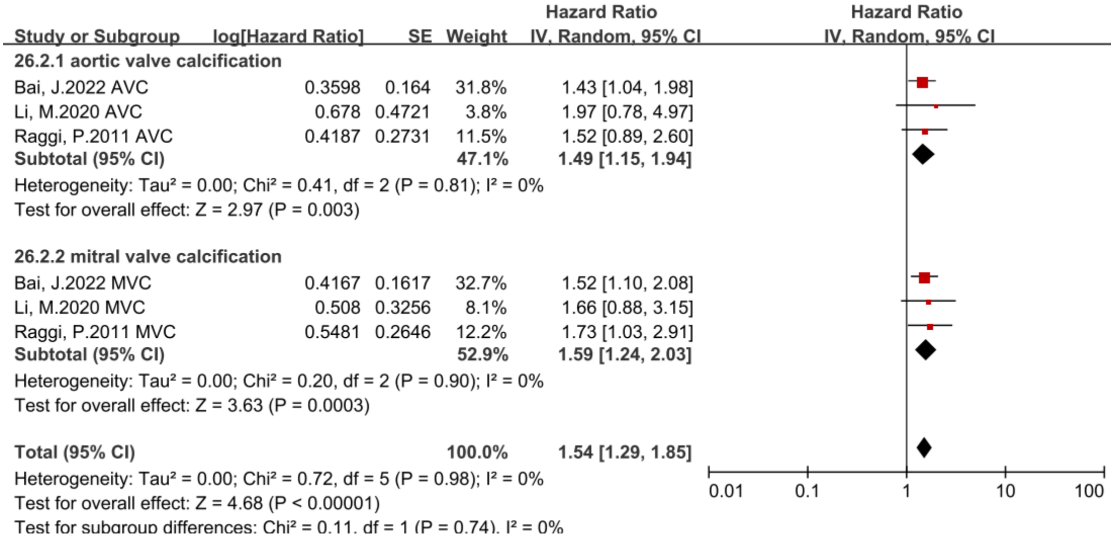

Supplementary Figure 4 Subgroup analysis for mortality according to the dialysis modality

Study or Subgroup	log[Hazard Ratio]	SE	Weight	Hazard Ratio IV, Fixed, 95% CI
**26.3.1 hemodialysis**				
Bai, J.2022 AVC	0.3598	0.164	21.6%	1.43 [1.04, 1.98]
Bai, J.2022 MVC	0.4167	0.1617	22.3%	1.52 [1.10, 2.08]
Chen, X. N.2015	0.4466	0.458	2.8%	1.56 [0.64, 3.84]
Li, M.2020	0.7706	0.3525	4.7%	2.16 [1.08, 4.31]
Li, M.2020 AVC	0.678	0.4721	2.6%	1.97 [0.78, 4.97]
Li, M.2020 MVC	0.508	0.3256	5.5%	1.66 [0.88, 3.15]
Panuccio, V.2004	0.0953	0.2454	9.7%	1.10 [0.68, 1.78]
Raggi, P.2011 AVC	0.4187	0.2731	7.8%	1.52 [0.89, 2.60]
Raggi, P.2011 MVC	0.5481	0.2646	8.3%	1.73 [1.03, 2.91]
**Subtotal (95% CI)**			**85.3%**	**1.51 [1.29, 1.78]**
Heterogeneity: Chi² = 3.48, df = 8 (P = 0.90); I² = 0%				
Test for overall effect: Z = 5.00 (P < 0.00001)				
**26.3.2 peritoneal dialysis**				
Shen, A.2021	0.6054	0.285	7.2%	1.83 [1.05, 3.20]
Yang, X.2018	0.0488	0.2771	7.6%	1.05 [0.61, 1.81]
**Subtotal (95% CI)**			**14.7%**	**1.38 [0.93, 2.03]**
Heterogeneity: Chi² = 1.96, df = 1 (P = 0.16); I² = 49%				
Test for overall effect: Z = 1.61 (P = 0.11)				
**Total (95% CI)**			**100.0%**	**1.49 [1.28, 1.73]**
Heterogeneity: Chi² = 5.63, df = 10 (P = 0.85); I² = 0%				
Test for overall effect: Z = 5.23 (P < 0.00001)				
Test for subgroup differences: Chi² = 0.19, df = 1 (P = 0.66), I² = 0%				

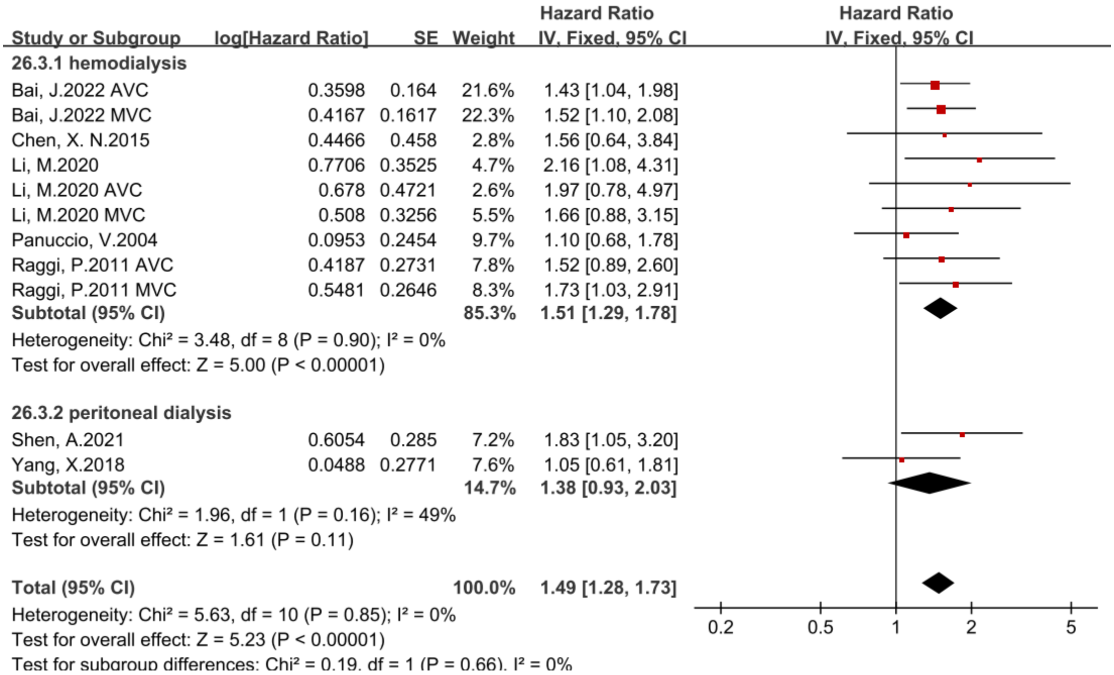

Search strategy

Search strategy for PubMed:

(((((((chronic kidney disease) OR (end-stage renal disease)) OR (hemodialysis)) OR (peritoneal dialysis)) AND (cardiac valve calcification))) AND (mortality)) AND (risk factor)

Search strategy for Embase:

#1 ((chronic AND kidney AND disease:ab,ti OR chronic) AND kidney AND insufficiency:ab,ti OR chronic) AND renal AND failure

#2 (((heart AND valve AND calcificatiion:ab,ti OR cardiac) AND valve AND calcification:ab,ti OR cardiac) AND valvular AND calcification:ab,ti OR heart) AND valvular AND calcification

#3 mortality

#4 risk AND factors

#5 #1 AND #2 AND #3 AND #4

Search strategy for Web of Science

((((TS=(chronic kidney disease)) OR TS=(end-stage renal disease)) AND TS=(cardiac valve calcification)) AND TS=(mortality)) AND TS=(risk factor)
